# Eosinophilia in school-going children with *Plasmodium falciparum* and helminth infections in the Volta Region of Ghana

**DOI:** 10.11604/pamj.2021.38.277.17379

**Published:** 2021-03-17

**Authors:** Verner Ndudiri Orish, Jones Ofori-Amoah, Kokou Hefoume Amegan-Aho, Emmanuel Udochukwu Osisiogu, James Osei-Yeboah, Sylvester Yao Lokpo, Emmanuel Alote Allotey, Joseph Adu-Amankwaah, Daniel Edem Azuma, Percival Delali Agordoh

**Affiliations:** 1 Department of Microbiology and Immunology, School of Medicine, University of Health and Allied Sciences, Ho, Volta Region, Ghana,; 2 Department of Pharmacology and Toxicology, School of Pharmacy, University of Health and Allied Sciences, Ho, Volta Region, Ghana,; 3 Department of Paediatrics, School of Medicine, University of Health and Allied Sciences, Ho, Ghana,; 4 Department of Science Laboratory Technology, Wa Polytechnic, Wa, Ghana,; 5 Department of Medical Laboratory Sciences, School of Allied Health Sciences, University of Health and Allied Sciences, Ho, Ghana,; 6 School of Medicine, University of Health and Allied Sciences, Ho, Volta Region, Ghana,; 7 Department of Nutrition and Dietetics, School of Allied Health Sciences, University of Health and Allied Sciences, Ho, Ghana

**Keywords:** Eosinophilia, school children, *Plasmodium falciparum*, parasitic infection

## Abstract

**Introduction:**

eosinophilia is seen in children infected with parasitic organisms. This study aimed at evaluating eosinophilia in children infected with Plasmodium falciparum, Schistosoma haematobium and intestinal helminths in the Volta Region of Ghana.

**Methods:**

five hundred and fifty primary school children were selected for this study from 5 primary schools in 2 districts and a municipal area of the Volta Region of Ghana. Blood, stool and urine samples were obtained and screened for P. falciparum, intestinal helminths and S. haematobium respectively. Socio-demographic information were obtained using a standardized questionnaire administration. Pearson chi square analysis was used to evaluate the association between eosinophilia and parasitic infections, and multivariate logistics regression analysis was used to identify factors independently associated with increased risk of eosinophilia.

**Results:**

a total of 145(26.36%) children had eosinophilia of which 107(73.79%) were infected with P. falciparum infection, (p=0.016); 18(12.41%) with S. haematobium infection, (p=0.016); and 3(2.07%) children were infected with intestinal helminth, (p=0.36). Children infected with P. falciparum had 2 times increased risk of eosinophilia (AOR=2.01, 95% CI, [1.29-3.2], p=0.02); while children from Davanu primary school had 4 times increased risk of eosinophilia (AOR=4.3, 95% [2.41-10.10], p<0.001).

**Conclusion:**

there was significantly high prevalence of eosinophilia among children infected with P. falciparum infection. A longitudinal study is needed to further understand the immune response of these children to parasitic infections.

## Introduction

Eosinophil is a granulocytic leucocyte responsible for immunity against parasites and mediation of allergic reactions in humans [1]. It constitutes about 1- 5% of the total white cell count, and a very effective component of innate immune system [2]. An abnormally high eosinophil count in the blood is termed eosinophilia; defined as eosinophil count greater than 450 cells/μL [3]. Eosinophilia is typically seen in adult and children with helminths and *Plasmodium falciparum* infections [4]. *Plasmodium falciparum* infection is a very common infection in malaria endemic areas of the world, affecting many, especially pregnant women and children [5]. The immunity against *P. falciparum* is orchestrated by a timely relay between T-helper cell type 1 (Th-1) and T-helper cell type 2 (Th-2) immune responses. The Th-2 immune response stimulates the production of eosinophils, especially in patients who are recovering from *P. falciparum* infection or those with uncomplicated infections [6, 7].

Helminth infections are very common in developing countries of the world, especially in sub-Saharan Africa where poor environmental sanitation and inadequate water is prevalent [8]. Intestinal helminths like Ascaris lumbricoides, Hookworm, *Trichuris trichiura*, and Trematodes like *Schistosoma haematobium* and mansoni are very common infections of children in endemic areas [8, 9]. Eosinophils are very crucial in the defense against helminths as they are produced through Th-2 immune response to larvae permeation of the human tissues [4, 10]. In Ghana, helminth and *P. falciparum* infections are endemic, causing significant morbidity and mortality in children [11-13]. It will be very important to evaluate the leucocyte profile of these children in a bid to further understand the immune modulation of these parasitic infections. The aim of this study therefore, was to evaluate the eosinophil count and identify risk factors in school-going children infected with helminth and *P. falciparum* in the Volta Region of Ghana.

## Methods

A total of 550 primary school children, aged 6-14 years, from 5 primary schools were enrolled for this study. The primary schools were purposively selected from 2 districts and a municipal area in the southern part of the Volta Region of Ghana. They included Freetown Primary School from the Ho municipality (the administrative capital of the region), Evangelical Presbyterian (EP) Primary Schools in Afegame and Kpetoe in the Agotime-Ziope district, and Dave and Davanu Primary Schools in the Adaklu district.

The study procedure has been previously described elsewhere [14, 15]. Briefly, blood, stool and urine samples were obtained from children who gave assent and whose parents consented to the study. Questionnaire was used to capture socio-demographic data of the children. Samples collected were transported in an ice chest to the laboratory for same day analysis. Laboratory diagnosis of malaria was done using *P. falciparum* specific Bioline SD rapid diagnostic test (RDT) (Standard Diagnostics, INC., South Korea), and Giemsa-stained thick and thin film preparations. *P. falciparum* infection was defined as a sample positive for either RDT or microscopy. Stool samples were analyzed using the wet mount technique for the detection of ova of helminthes. Urine centrifugation method was used for the detection of S. haematobium ova.

A complete blood count analysis was done on the blood samples using automated haematology analyzer (Sysmex, Kakogawa, Japan). Haemoglobin concentration and eosinophil counts were obtained from the complete blood profile. Anaemia was classified into severe (Hb<7g/dl), moderate (7-9.9g/dl), and mild anaemia (<11g/dl) [15]; while eosinophilia was classified as eosinophil count greater than 450 cells/μL [3].

Statistical analysis was done using IBM SPSS Statistics version 21.0 (IBM Corporation, Armonk, NY, USA). Frequency distribution was performed for all variables of interest. Pearson chi square tests analysis was used to investigate the association between eosinophilia and other variables. Multivariate logistic regression was then used to investigate factors independently associated with increased risk of eosinophilia among the children. Analyses were done with 95% confidence interval (CI), and p value of 0.05 and below was considered statistically significant.

## Results

A total of 145(26.36%) children had eosinophilia; 383(69.64%) were positive for *P. falciparum*, 22(4.00%) had intestinal parasitic infections (i.e. Ascaris lumbricoides [7], Hookworm [5], Entamoeba spp (10), whilst 101 (18.36%) children were anaemic (Table 1). A significant proportion of the children infected with *P. falciparum* had eosinophilia (107, 73.79%, p=0.016) than those who were uninfected (Table 1). Also, children infected with S. haematobium infection were less eosinophilic, however this finding was not significant (15, 12.41%, p=0.16) (Table 1). Davanu Primary School in the Adaklu district significantly had more children with eosinophilia (42, 28.98%, p<0.001) (Table 1). Majority of the children with eosinophilia had normal haemoglobin concentration (106, 73.10%), although this finding was not significant (p=0.2).

**Table 1:** characteristics of children stratified by eosinophilia status

Characteristics	Eosinophilia N=145 (%)	Normal N=405 (%)	Total N=550 (%)	P value
***Plasmodium falciparum***				
Positive	107(73.79)	276(68.15)	383(69.6)	0.016
Negative	38(26.21)	129(31.85)	167(30.3)	
**Schistosoma haematobium**				
Positive	18(12.41)	39(9.63)	57(10.36)	0.16
Negative	127(87.59)	366(90.37)	493(89.6)	
**Intestinal helminth**				
Present	3(2.07)	19(4.69)	22(4.00)	0.36
Absent	142(97.93)	386(95.31)	528(96.0)	
**Haemoglobin level**				
Anaemia	39(26.90)	62(15.30)	101(18.3)	0.24
Normal	106(73.10)	343(84.70)	449(81.6)	
**Primary school**				
Dave	21(14.49)	63(15.56)	84(15.27)	<0.001
Freetown	31(21.35)	94(23.21)	125(22.7)	
Davanu	42(28.98)	37(9.14)	79(14.36)	
EP Afegame	29(20.00)	94(23.21)	123(22.3)	
EP Kpetoe	22(15.18)	117(28.88)	139(25.2)	

Data is presented as frequency with corresponding percentage in parenthesis. P is significant at 0.05

**Figure 1:**
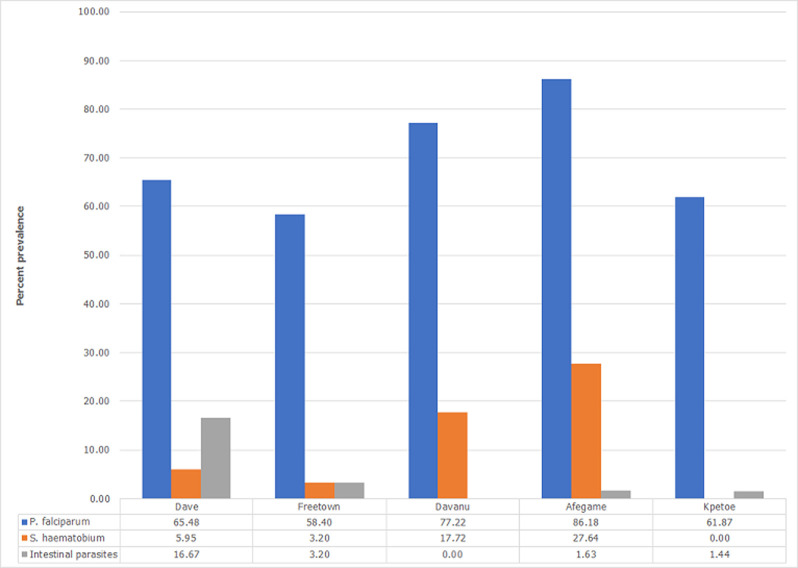
percentage prevalence of parasitic infections among children in the 5 primary schools

A significant proportion of the children from Afegame E.P Primary School in Agotime-Ziope district (106, 86.14%, p=0.001), and Davanu Primary School in Adaklu district (61, 77.22%, p=0.001), were infected with *P. falciparum* infection (Figure 1). Though not statistically significant (p=0.16), Davanu (14, 17.72%) and Afegame E.P (34, 27.64%) Primary Schools also recorded a high proportion of the children infected with S. haematobium (Figure 1).

Multivariate logistics analysis (Table 2) showed that children infected with *P. falciparum* were independently associated with increased risk of eosinophilia (Unadjusted OR, 2.11; 95% CI, 1.35-3.31; p=0.001; Adjusted OR, 2.02, 95% CI, 1.29-3.22; p=0.02). Children from Davanu Primary School was independently associated with higher risk of eosinophilia (Unadjusted OR, 4.83; 95% CI, 2.42-9.67; p<0.001; Adjusted OR, 4.39, 95% CI, 2.41-10.10; p<0.001).

**Table 2:** multivariate logistics regression analysis of the risk of eosinophilia among the primary school children

Variable	Unadjusted OR (95% CI)	p value	Adjusted OR (95% CI)	p value
**Gender**				
Male	1.35(0.88-2.05)	0.17	1.29(0.84-1.98)	0.25
Female	Ref			
**School**				
Dave prim	1.32(0.60-2.90)	0.49	1.48(0.627-3.24)	0.33
Freetown prim	1.86(0.94-3.69)	0.74	2.08(1.03-4.16)	0.06
Davanu prim	4.83(2.42-9.67)	<0.001	4.39(2.41-10.10)	<0.001
Afegame EP	1.52(0.75-3.08)	0.25	1.51(0.72-3.17)	0.28
Kpetoe EP	Ref			
***P.falciparum***				
Positive	2.11(1.35-3.31)	0.001	2.02(1.29-3.22)	0.02
Negative	Ref			
**S.haematobium**				
Positive	1.47( 0.77-2.8)	0.24	1.46(0.76-2.81)	0.25
Negative	Ref			
**Hemoglobin**				
<11g/dl	1.38(0.81-2.35)	0.24	1.48(0.86-2.55)	0.16
≥11g/dl	Ref			

OR=odds ratio, CI=confidence interval, Ref= Reference. P is significant at 0.05

## Discussion

The prevalence of eosinophilia as seen in this study (26.4%) is a fairly common manifestation among children living in *P. falciparum* and helminths endemic areas [4, 6, 10]. This study recorded only asymptomatic *P. falciparum* infected children (69.6%) with a significant proportion (73.8%) having elevated eosinophil count. Many studies have reported reduced eosinophil count in acute and complicated malaria, and eosinophilia in patients receiving treatment and recovering from malaria in uncomplicated and asymptomatic infections [6, 7, 16, 17]. This fluctuation of the eosinophil count, in relation to the severity and intensity of *P. falciparum* infection, vis-a-vis the low levels in acute infections with a progressive increase in recovering patients, indicates a healthy immune response underscoring the anti-plasmodial activity of eosinophils [6, 7]. As infection resolves with elevated eosinophil count, other haematological parameters like haemoglobin concentration also improves [7]. This might explain why majority of the children with eosinophilia (73%) in this study had normal haemoglobin concentration.

There was a less remarkable prevalence of eosinophilia among children infected with S. haematobium (12%) and intestinal helminths (2%). Studies have reported eosinophilia among children with S. haematobium and intestinal helminth infections [10, 18-20]. However, eosinophilia tend to be lowered following treatment with anti-helminthic drugs and periodic deworming exercises [20, 21]. This might explain the reason for the low prevalence of eosinophilia seen in children with helminth infection, because of the regular periodic deworming exercise initiated by the School Health and Education Programme (SHEP) of the Ghana Education Service/Ministry of Education and the NTDCP of the Ghana Health Service/Ministry of Health [22, 23]. Praziquantel and albendazole are administered to target S. haematobium and intestinal helminths through these initiatives, respectively [23].

Children from Davanu Primary School had the highest prevalence of eosinophilia (28.9%). These children had a significantly high prevalence *of P. falciparum* infection as well. This high prevalence of *P. falciparum* infection might be responsible for the high prevalence of eosinophilia seen among these children. Another factor that might have been responsible for this high prevalence of eosinophilia is the rural setting of Davanu Primary School. Davanu is located in the Adaklu district, which is a predominantly rural settlement with poor water supply and inadequate sanitation [24]. These conditions are very much linked to higher risk of other various parasitic infections, allergies and skin diseases that can cause eosinophilia [19, 25].

## Conclusion

This study showed that there was significant high prevalence of eosinophilia among children infected with *P. falciparum* infection. Primary school children from deprived rural community had significantly high eosinophilia. Further studies are needed to fully understand the dynamics of parasitic infections, deworming and eosinophilia among these children.

### What is known about this topic

- Eosinophil is a granulocytic leucocyte responsible for immunity against parasites and mediation of allergic reactions in humans;
- Abnormally high eosinophil counts in the blood is termed eosinophilia and is defined as eosinophil count greater than 450 cells/μL;
- Eosinophilia is typically seen in adult and children with helminths and Plasmodium falciparum infections.

### What this study adds

- This study further supports the finding of eosinophilia in P. falciparum as there was significant high prevalence of eosinophilia among children infected with P. falciparum infection;
- The study demonstrates that the prevalence of eosinophilia is higher among children in the rural areas than among their urban counterparts.
